# A systematic review of the use of adolescent mystery clients in assessing the adolescent friendliness of health services in high, middle, and low-income countries

**DOI:** 10.1080/16549716.2018.1536412

**Published:** 2018-11-28

**Authors:** Venkatraman Chandra-Mouli, Cosima Lenz, Emmanuel Adebayo, Iliana Lang Lundgren, Lucia Gomez Garbero, Subidita Chatterjee

**Affiliations:** a Department of Reproductive Health and Research, Human Reproduction Programme, World Health Organization, Geneva, Switzerland; b UCLA Fielding School of Public Health, Los Angeles, USA; c Adolescent Health Unit, Institute of Child Health, University of Ibadan, Ibadan, Nigeria; d Department of Health Policy and Management, Globa lGiving Foundation, Georgetown University - Women’s and Gender Studies; e School of Psychology, Universidad Catolica del Uruguay; f Independent expert, Kolkata, India

**Keywords:** Youth friendly services, adolescent sexual and reproductive health, participatory research methods

## Abstract

**Background**: Mystery client methodology is a form of participatory research that provides a unique opportunity to monitor and evaluate the performance of health care providers or health facilities from the perspective of the service user. However, there are no systematic reviews that analyse the use of mystery clients in adolescent sexual and reproductive health (ASRH) research and monitoring and evaluation of programmes.

**Objective**: To assess the use of adolescent mystery clients in examining health care provider and facility performance in providing ASRH services in high, middle, and low-income countries.

**Methods**: We carried out a systematic review of published journal articles and reports from the grey literature on this topic from 2000 to 2017 (inclusive). Thirty research evaluations/studies were identified and included in the analysis. We identified common themes through thematic analysis.

**Results**: The findings reveal that researchers and evaluators used mystery client methodology to observe client-provider relationships, and to reduce observation bias, in government or private health facilities, NGOs, and pharmacies. The mystery clients in the evaluations/studies were young people who played varying roles; in most cases, they were trained for these roles. Most reported good experiences and friendly providers; however, some reported lack of privacy and confidentiality, lack of sufficient written/verbal information, and unfavourable experiences such as sexual harassment and judgmental comments. Female mystery clients were more likely than males to report unfavourable experiences. Generally, the methodology was considered useful in monitoring and evaluating the attitudes of health service providers and ASRH service provision.

**Conclusions**: The research evaluations/studies in this review highlight the usefulness of mystery clients as a method to gain insight, from an adolescent perspective, on the quality of ASRH services for research and monitoring and evaluation of programmes.

## Background

Mystery client (MC) methodology is a form of participatory research that provides a unique opportunity to monitor and evaluate the performance of health care providers or health facilities from the perspective of the service user [1]. MC methodology has been used in health research for more than 20 years [2,3], and Gonsalves and Hindin’s recent review on youth access to SRH care in pharmacies includes studies that involved mystery clients [4]. However, there are no systematic reviews published to our knowledge that analyse the uses, advantages, and disadvantages of MC methodology in adolescent sexual and reproductive health (ASRH) research and monitoring and evaluation (M&E) of programmes. This review focuses on describing the use of adolescent MCs in examining adolescent health service provision.

While this methodology has been used among adults and adolescents alike [5–8], in this review we consider the use of adolescent MCs to assess the friendliness of adolescent health services across high-, middle-, and low-income countries. ASRH services vary by type of care provided, cadres of health care providers or healthcare facilities (i.e. hospitals, clinics and pharmacies), and thus this review includes evaluations/studies that cover a range of contexts, including but not limited to family planning services, services for sexually transmitted infections (STIs) including Human Immunodeficiency Virus (HIV), and pharmacy services in formal and informal settings.

In this review, we set out to answer the following questions:

1. Why was the MC methodology used in the evaluation/study of ASHR services?
2. What were the roles assigned, how were the MCs prepared, and what methods and tools were used to carry out the assessment?
3. What types of healthcare facilities and cadres of health care providers were examined with MCs?
4. What were the principal findings from the methodology in the evaluations/studies?
5. What were the overall perceptions of the use of MCs in assessing health care provider and facility performance?

## Methods

### Data collection

Though no formal protocol was developed for this research, we followed the PRISMA guidelines to perform a systematic review of the literature, including journal articles and grey literature, issued between the years of 2000 and 2017 (inclusive) that used adolescent MCs as part of their methodology to assess ASRH health care provider and health facility performance [9]. We sought evaluations/studies published in English and Spanish across low-, middle-, and high-income countries that used the MC methodology as part of their study to understand adolescents’ access to ASRH health care.

We conducted the literature search with Google, Google scholar, POPLINE, PubMed, GIFT, JSTOR, MEDLINE, and EMBASE. The original literature review was conducted in 2015, and in 2017 the paper was updated and expanded upon with an additional literature search. The review helped identify organizations that used the MC methodology, which included the Program for Appropriate Technology in Health (PATH), the USA Agency for International Development (USAID), Pathfinder International, the United Nations Population Fund (UNFPA), and Advocates for Youth. Table 1 describes the complete search strategy for this review, including the keywords and hand searches used to find the relevant publications. An initial abstract screening, followed by a full text screening was conducted. The full text was screened in case that the information presented in the abstract was not sufficient to make a conclusive decision. Authors were only contacted in the cases that their studies/evaluations were inaccessible online. The search yielded over 1000 publications, and we screened the publications by title, abstract, and full paper in cases where the abstracts were unclear. We identified 51 total research evaluations/studies from this screening and the additional update in 2017.

**Table 1.** Search protocols.

**Databases**
Google, Google scholar, POPLINE, PubMed, GIFT, JSTOR, MEDLINE, EMBASE
**Hand Searches**
Use of mystery clients to assess adolescent health worker behaviour, mystery client used to evaluate ASRH, mystery client adolescent health, mystery shopper adolescent health, adolescent undercover patients, young people mystery clients, mystery client youth
**Search Terms**
*Mystery clients terms*
Mystery clients, mystery shoppers, mystery patients, simulated patients, undercover patients, dummy patients, undercover consumers
*Adolescent terms*
Adolescents, young people, youth, teenagers
*Health service terms*
Sexual and reproductive health service, sexual health, reproductive health, ASHR, contraceptive services, contraction, family planning, condoms, oral contraception, pharmacy services, accessibility, health worker behaviour, provider attitudes, provider behaviour, client satisfaction

### Data analysis

**Figure 1.**
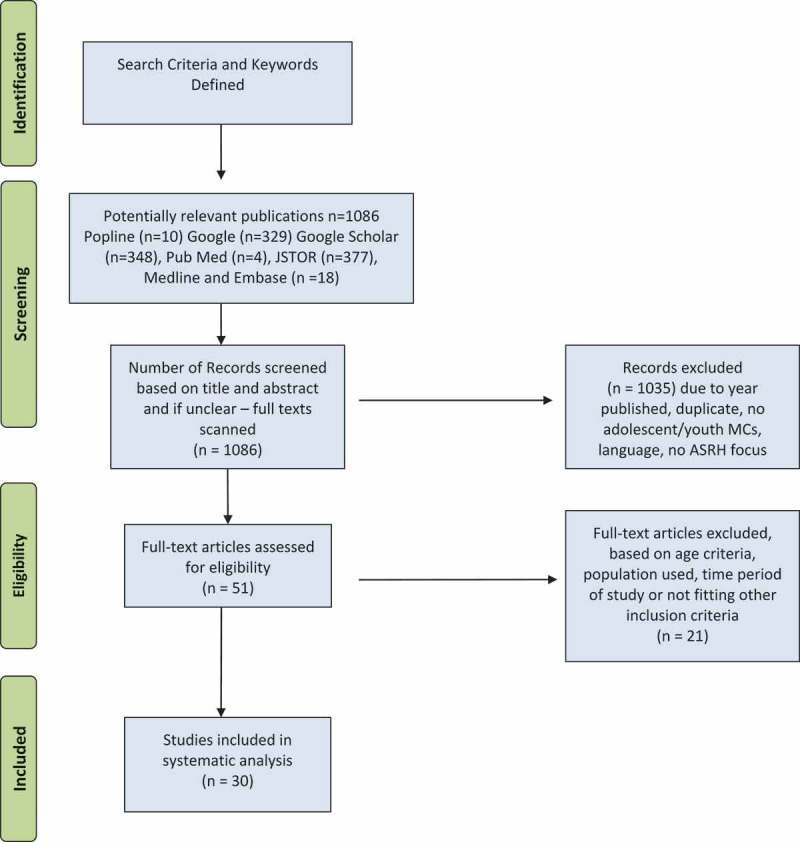
PRISMA flow diagram.

We used a list of inclusion criteria Table 2 to review identified publications for their validity. Of the 51 publications identified in the initial screening, 21 were excluded because they did not align with these criteria. Although the inclusion criteria initially included articles in Spanish and English, no articles in the Spanish language were finally included in this paper as they did not meet the other criteria or because documents reporting on the research could not be found. Two independent reviewers carried out the literature review, and consulted with a third party researcher regarding disagreements. The PRISMA flow diagram Figure 1 organizes the details of our search.

**Table 2.** Inclusion criteria.

Time frame	2000–2017
Study Population	Focused on service provision for young people (10–24), male or female
Study Design Methodology	Utilized quantitative and/or qualitative methods Included an evaluation portion using adolescent MCs (males or females, aged 10–24) MCs assessed health provider (nurse, doctor, pharmacists, technician, staff, receptionist) behaviour when delivering ASRH care
Geographic Scope	*Low and low-middle income countries* – (African, South East-Asian, Latin and South American, Western-Pacific, Eastern-Mediterranean Regions) *High-income countries* – (USA, Western Europe)
ASRH care	Focusing on how adolescents perceived or observed health provider behaviour during mystery client visits to service providers of ASRH care
Language	Published in English or Spanish
Article type	Peer-reviewed journal articles (evaluations/studies), grey literature

We analysed all evaluations/studies with a framework based on six principal criteria: i) overall objective and methodology; 2) justification of the use of MC methodology; 3) the role of MCs and the training/support they received; 4) the setting and type of health workers assessed; 5) principal findings; and 6) perceptions on the use of MCs. The 30 evaluations/studies were thereafter thematically analysed for shared methods used, justifications, and common findings. Thematic analysis consisted of intently sorting studies/evaluations based on shared characteristics including types and certain elements such as training MC in their methodologies, analytical methods, reasons for using MC, and outcomes of having used MCs.

An analysis of the quality of the evaluations/studies was beyond the scope of this paper as the methodology was used in different contexts. While some used the methodology as an evaluation method for particular programmes, others reported using it as a research method to help design and develop interventions. The intentional lack of incorporation of a quality assessment stemmed from the aim of the analysis focusing on the types of MC methodologies and outcomes recorded, which would not have accurately reflected the available information if studies were excluded based on quality. The small scale, lower quality status of some studies is recognised and further reflected upon in the limitations section.

## Context

The involvement of young people in research processes is an important part of planning and implementation of programmes and interventions. Adolescents and young people have increasingly been involved in research at different stages; planning, implementation, and M&E [10]. Researchers no longer view adolescents as just subjects, but as equal partners and contributors in research planning and implementation [10,11]. There has beengrowing calls for the participation of young people in programme and policy development [12], and the MC method provides an excellent opportunity.

In the MC method, participants take on a role other than themselves when receiving health services. The health care providers are typically unaware of the undercover nature of the client and thereby allow the client to observe their natural behaviour [13]. MC methodology is considered a participatory research method as it fulfils the criteria set out by Cornwall and Jewkes [14] and it ‘emphasises participation and action’ [14] while encouraging the involvement of ‘people whose actions and worlds are under study’ [11]. It provides important insight from the user’s perspective of health services or other programmes. MCs are also referred to as simulated patients, undercover patients, or mystery shoppers [13].

Critiques of the methodology include difficulties in recruitment and training of MCs, recall bias, reliability of information provided by the MCs, and the limitations to the type of information that can be collected [1]. However, research has continued to affirm its effectiveness and value [15].

## Results

**Figure 2.**
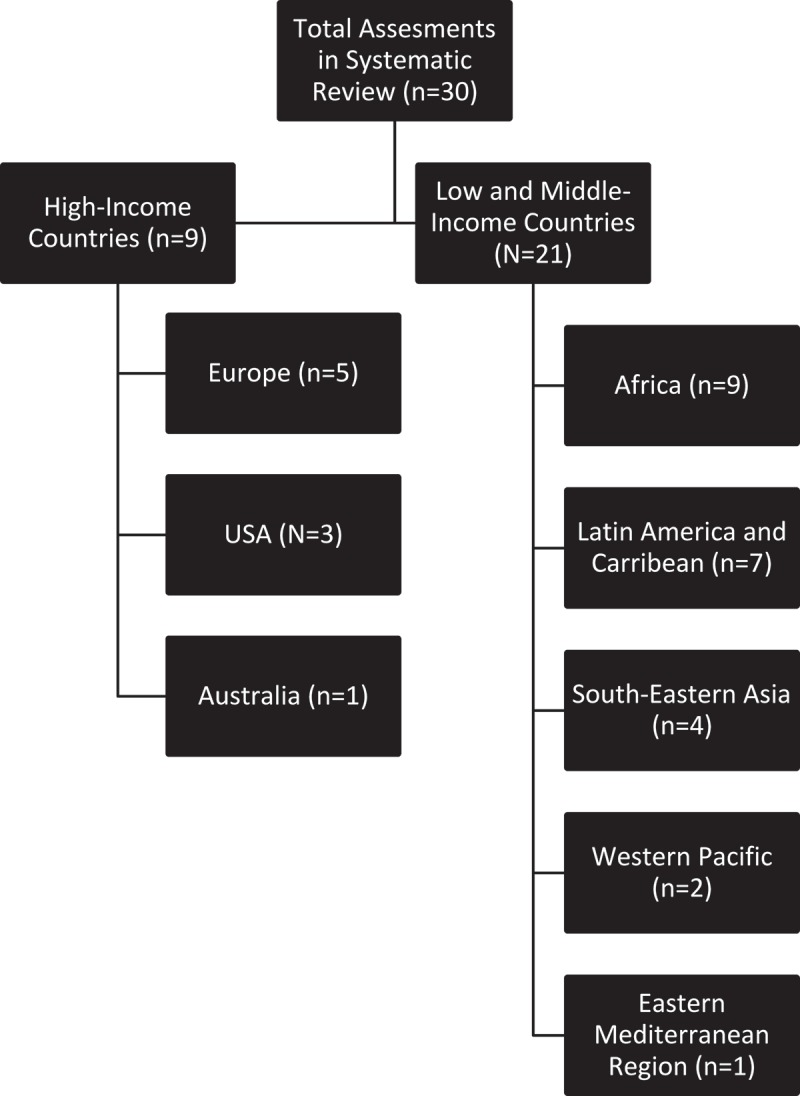
Geographic distribution of studies.

Our findings and conclusions are based on the final selection of 30 evaluations/studies. Nine of these (30%) were conducted in high-income countries and 21 (70%) in low- and middle-income countries. The geographical distribution of the evaluations/studies in this systematic review is displayed in Figure 2. Of the 30 evaluations/studies, 18 were journal publications and 12 were grey literature publications. Of the 18 published articles, 12 were from low- and middle-income countries and nine were from high-income countries

### Use of MC methodology

Fourteen of the 30 evaluations/studies reviewed used the MC approach as the only methodology whereas 16 applied a mixed methodology approach. Of the mixed method evaluations/studies, 11 used interviews to elicit information from varying respondents that included health service providers, adolescents, and opinion leaders within the community such as senior staff, six used questionnaire-based surveys, and five used focused group discussion among adolescents, young people, health service providers and other stakeholders such as teachers and local residents. There were no observable differences in methodologies used by high-income and middle- or low-income countries.

The performance of health providers and health service provision facilities were reviewed in all cases, but the types of providers and services varied. MCs were used to assess staff and services at pharmacies in 10 evaluations/studies [16–25], and health service providers at reproductive health centre/clinics in two evaluations/studies [26,27]. Nine evaluations/studies assessed services and/or service providers in mixed settings including hospitals, local health facilities, dispensaries, and NGOs [28–36], while six looked at community health facilities or clinics [37–42] with one focusing on teaching hospitals [40].

Two evaluations targeted the youth friendliness of services for the Lesbian, Gay, Bisexual and Trans (LGBT) population [26,43], one in a high-income and one in a low-income country. Six of the evaluations/studies aimed to assess the youth friendliness of the services and providers in their study [16,26,29,35,40,44]. Nine evaluations/studies also examined the effects of the implementation of training for service providers or youth friendliness programmes [16–18,25,28,34,42,44,45].

## Key findings

### Why was the MC methodology used in the evaluation/study of ASHR services?

The justification for using MCs varied among the evaluations/studies; however they can be grouped with similar explanations. The most common reason for the use of MCs was to assess/observe the interaction between service providers and patients, with particular interest in the friendliness of services, from the perspective of adolescents [16,30,35,39,40]. Another reason was to reduce or eliminate bias [13,17,18,21–25,29,33,36]. By using trained MCs, the researchers and evaluators intended to avoid observation bias or bias from the Hawthorne effect, whereby the observer could observe the subjects without their knowledge of the observation to influence their behaviour. Training helped to ensure that the mystery clients already knew what to look for in health care providers/facilities. Therefore, the evaluations/studies could use the methodology to understand factors that motivated or discouraged adolescents and young people from accessing health services.

### What were the roles assigned, how were the MCs prepared, and what methods and tools helped carry out the assessment?

#### Roles of MC

Our review found that while some methodologies reported specific roles to be played by the MCs during health care visits, others had less formal or undefined roles.

Six of the reviewed evaluations/studies did not provide formal roles for their MCs. In these cases, MCs were asked to assess ease of access to health facilities, the environment of health facilities, staff and health provider behaviour, and service provision or specific aspects of care, such as quality of advice received [7,17,18,31,38,44]. Twenty-one of the reviewed evaluations/studies provided their MCs with specific scenarios, with either a script or a specific question or concern to ask during the medical visit [17,19,21–25,27,29,32–35]. Two of the reviewed evaluations/studies provided MCs with scripted scenarios, but failed to include information on what the scenarios or roles entailed [16,26,36].

Thirteeen of the 30 reviewed evaluations/studies, recruited both male and female adolescents or young people as MCs to assess health provider behaviour and services [16,20,25–27,32,35–37,39,41,44]. One study recruited only male MCs [45], and only one study included non-binary gendered MCs in their study design [26]. Five evaluations/studies did not specify sex of the MCs in the description of the methods [28,31,38,40,43]. Eleven of the 30 evaluations/studies reviewed did not specify the ages of the MCs recruited for the study [17,19,20,30,32,33,35,38,40,41,43], while the rest recruited individuals who were 25 years old and under [16,18,21–29,31,34,36,37,39,44,45].

Adolescent MC roles in the reviewed evaluations/studies included: (I) young females seeking pregnancy prevention or contraception/emergency contraception services [13,15–18,21–26,29,30,32,34], (II) females seeking counselling on premarital sex [29,35], (III) males seeking condoms [27,29,32,36,37,44], (IV) males and females seeking information or treatment for STIs [29,35,39], (V) or HIV testing and information [20,37,42], (VI) females requesting information about menstrual and associated problems [30], and (VII) seeking counselling for unwanted pregnancy or abortion [33,35].

Three evaluations/studies did not clearly specify the scenarios the MCs were to play [3,14,41]. The first of these stated that the MCs were to have a reproductive health complaint [14], the second stated that the MCs were to enact scenarios and did not provide further context [3], and the last trained their MCs to present critical SRH scenarios to the health care providers [41].

#### Training/preparation of MC

Some evaluations/studies detailed more in-depth training and support sessions than others in preparing the MCs for their roles to assess healthcare provider behaviour and service quality. Twenty-three of the reviewed evaluations/studies described a training module to prepare the MCs for their roles prior to their healthcare centre visits. Some studies included methods that provided education on SRH in addition to information on the MC roles they were to play [11,16,20,26,28,30–34,37,40,42,45]. Seven indicated that their trainings also incorporated information related to specific or general evaluation techniques [16,20,26,28,31,37,45]. One of the reviewed studies adopted preparation methods from *Mystery Shopping in Sexual Health: A Toolkit for Delivery* [18]. In some of the reviewed evaluations/studies, training sessions involved learning the specific scenarios MCs were expected to role play [17,21–25,27,29,35,36,39,44].

#### MC assessment tools

The evaluations/studies described an array of methods for data collection from the MCs’ visits. After the undercover visits, six of the evaluations/studies used questionnaires to debrief the MCs [23,28,30,32–34] and six interviewed MCs [97, 28, 37, 41, 42, 44]. Two studies utilized both interviews and questionnaires as a debriefing technique [36,39] and one study used a checklist to gather information from the MC post visit [45]. Four of the reviewed evaluations/studies described that MCs filled out observation reports [17,27,31,35] and one study had them complete a survey [16]. Other data collection methods included reflection sessions [19,26] and a rating system completed by the MCs [43]. Seven of the reviewed studies did not elaborate on the method of debriefing used [18,21,22,24,25,38,40].

Three of the reviewed evaluations/studies recorded information for data collection; in one evaluation the MCs brought digital recorders into the visit with them [29] while in the other two studies the MCs’ debrief interviews were digitally recorded [30] and filmed [28].

The reviewed evaluations/studies described a variety of data analysis techniques based on the type of data collected and the collection method. The majority of the evaluations/studies had research staff and coordinators analyse the data, while only one study specified the involvement of youth in the data analysis [27]. Five of the reviewed evaluations/studies used thematic analysis or coding [17,27,29,31,32]. Twelve of the reviewed evaluations/studies statistically analysed their quantitative data with a range of methods, such as regression analysis on platforms including SASS and Stata [19,21,22,24,25,33,34,36,42,44,45]. Four of the reviewed evaluations/studies used both statistical and thematic analysis methods [23,37,39,41]. Two employed a rating or score based analysis [35,36], and one analysed their audio-recorded MCs’ debrief manually; however, the last did not provide additional information [30]. Six studies did not include information on data analysis methods [16,18,20,26,28,38,40].

### What types of healthcare facilities and cadres of health care providers were examined with MCs?

MC methodology was used in diverse settings (Tables 3 and 4) including (I) Government or public facilities (including secondary and primary health care centres), (II) private health facilities, (III) adolescent health service delivery centres, (IV) facilities run by faith-based organizations, (V) NGO facilities, (VI) FP clinics/reproductive health clinics, and (VII) private and public pharmacies.

**Table 3.** Evaluations and studies in high income countries.

Country, Publication	Objective and Setting	Methods	Role, Training, and Support of MC’s	Findings and Impressions of MC use
**Scotland**; MacGinty and Marriot, 2014 [26]	To explore accessibility, youth friendliness, and LGBT awareness of services in youth sexual health centers	Criteria and reflection sessions to assess MC experiences	12 male, female, and non-binary gendered MCs aged 16–25 Assessed services, waiting area, staff, and access to resources with scenarios approved by lead worker Training included education on sexual health, ethics, evaluation criteria, and team building	Most staff and clinics were welcoming Most personnel used correct gender pronouns and had some knowledge of transgender issues Some providers assumed clients’ sexual activity and knowledge Some youth had to reveal their sexual orientation to providers Not all facilities had LGBT posters/pamphlets
**UK**; Council NC, 2015 [28]	To evaluate the views of young people on health services (GP practices, sexual health centres, pharmacies) in Nottinghamshire	MCs assessed health service website content, information in health settings/reception areas, and information via phone calls to health providers MCs completed questionnaires and filmed their feedback	20 MCs aged 14–19 Assessed access, clinic environment, confidentiality, staff, and quality of information Training included education on health issues, young people’s rights and laws, confidentiality, and evaluation techniques	Many services categorized as ‘good’ Bad experiences with unfriendly staff, lack of appropriate information, and concerns with privacy/confidentiality Website content outdated and centers lacked posters targeting young people MC’s provided a wealth of feedback on the website and services
**UK**; NHS, 2012 [18]	To assess the design and development of a training program for pharmacists to improve contraception service delivery in pharmacies in South-East London due to high teenage pregnancy rates	Methodology for MC’s adapted from Mystery Shopping in Sexual Health: A Toolkit for Delivery [10]	7 female MCs aged 16–18 Evaluated 5 aspects of oral contraceptive service: advertising, confidentiality, friendliness of staff, quality of service, ability to receive contraception, and patient satisfaction	Lack of consistent information on OC Some found staff pleasant while others found staff unfriendly/unhelpful Most pharmacists were knowledgeable on OC and gave appropriate information Difficult to get an appointment in some areas due to staff shortages Posters on contraception services were not available in all clinics
**UK**; Sykes S. and O’Sullivan K., 2006 [28]	To evaluate the accessibility and quality of services provided by sexual health and counseling services for young people using the MC approach in Croydon, UK	MCs visited sites in pairs and used assessment forms to document experiences after visits Young people, alongside research workers, analyzed qualitative data and identified key themes	16 female and 3 male MCs aged 13–21 Assessed services using 4 scenarios: asking about free condoms, asking for advice on contraception options, asking for advice on options after discovering a pregnancy, and asking for advice on emergency contraception Training consisted of learning scenarios, creating standards for services when working with young people, and role-playing	Most service providers delivered the necessary services Challenges arose concerning a lack of explanation particularly on confidentiality Difficulties arose with staff in the reception area who expressed that the services were inappropriate for the young clients
**USA**; Sampson O et al., 2009 [19]	To assess the role of pharmacy access to EC in reducing unintended pregnancies in adolescents, focusing on language barriers. Assessed pharmacists and health workers at 115 pharmacies in rural and urban California.	Concurrent nested model- mixed method qualitative and quantitative study design Interviews with pharmacists and health providers on the provision of care to adolescents MCs called pharmacies asking for EC Calls coded as successful if EC was provided immediately, unsuccessful if language/service not available, and vague or specific if they were referred to another service generally or to a specific location Data analyzed through SAS and Pearson’s Chi-square test	3 female MCs assessed provision of EC by calling pharmacies in Spanish and English Two scenarios: a 15 year old female had unprotected sex the night before and an 18 year old female had unprotected sex 4 days prior. Each pharmacy received four calls: each scenario in English and Spanish	Spanish speaking patients were less likely to receive contraception than English speakers Clients calling rural pharmacies were less likely to receive contraception then calls to urban ones All clients reported that the staff didn’t initiate the use of an interpreter and in one situation staff laughed at the broken English of the client A client had to speak with a male janitor due to the unavailability of a translator in the facility Several clients were denied service because they were too young, needed parental permission, or the staff expressed it was an unacceptable method for the client
**USA**; Wilkinson et al., 2017 [21]	To compare availability and access of EC for female adolescents before and after removal of age restrictions	Female MCs posed as 17-year-old adolescents in need of EC Standardized scripts Visited 979 pharmacies in 5 US cities Also incorporated income levels of pharmacy neighborhoods in the analysis	2 research assistants posed as 17-year-old females Male MCs were not used	Didn’t analyze the value or use of MC’s but determined that the proportion of pharmacies indicating that EC was available did not vary by neighborhood income level and barriers to EC access have not changed from 2012 Despite removal of age restrictions, accuracy of information provided did not differ by neighborhood income or from previous study Correct information about access to EC provided 51.6% of the time
**Australia**; Hussainy et al., 2015 [22]	To determine ECP supply practices of pharmacies in Victoria after release of updated guideline	Mystery client telephone calls to 515 pharmacies including one scenario where a woman under age 16 requested ECP	3 telephone call scripts: client seeking access outside the 74 hour time frame, a woman seeking access under 16 years of age, and a woman seeking access for future use Training of MCs not extensively discussed Only one scenario applicable to adolescents.	For a woman under age 16: more than half agreed to supply ECP, and used the guideline to assess eligibility 5.6% were only willing to provide ECP if the women had a doctor’s prescription or recommendation. Value of us of MC’s not discussed in depth
**France**; Delotte et al.; 2008 [23]	To evaluate the delivery of EC to minors in French pharmacies	Adolescent MCs requesting ECPs in pharmacies to evaluate the privacy of the provision of the EC, information delivered on contraception or STIs, sex of the person who they interacted with at the pharmacy, delivery of the contraception, and the reason for refusal (if it was refused)	Four minors carried out an anonymous investigation in 53 randomized pharmacies in Nice Scenario: woman had not used a method of contraception during last intercourse Data collected electronically after the pharmacy visit	Oral contraception was refused to minors by 37.7% of pharmacies Few pharmacies explained the side effects and use of EC
**US**; Wilkinson et al.; 2012 [24]	To assess the accuracy of information provided to adolescents and their physicians when calling pharmacies to ask about EC	Female callers telephone 943 pharmacies in 5 US cities Used standardized scripts	Research assistants posed as 17 year old females or female physicians calling on behalf of 17-year-old patient Male MCs not used.	19% of pharmacies incorrectly told adolescents that it would be possible to obtain EC; 3% of pharmacies stated this to physician callers Adolescent callers put on hold more and spoke to self-identified pharmacists less often than physician callers Most pharmacies had EC in stock but misinformation about who can take it is common

**Table 4.** Evaluations and studies in low and middle income countries.

Country, Publication	Objective and Setting	Methods	Role, Training, and Support of MC’s	Findings and Impressions of MC use (if stated)
**South Africa**; Geary, R. S. et al 2015 [44]	Evaluate whether primary health facilities in the YFS (Youth Friendly Services) Program- provided better quality care than primary care facilities not in the program Assessed health workers at 15 primary health care clinics in Soweto, a town on the outskirts of Johannesburg	One male and one female MC visited each clinic (7 total MCs) Composite score for each clinic based on MC experiences highlighting staff interaction, consultation, privacy, confidentiality, health worker characteristics, and clinic environment Brief questionnaire following MC’s visits Data analysis: multilevel regression models	Male MCs: 22 years old and asked about the reliability of condoms, if they can break, and how to use them Female MCs: 22 years old and asked about pregnancy prevention, and information on contraception options besides condoms Training consisted of scenarios role-playing with research nurses, who aided in the development of the scenarios	Primary care facilities part of YSF didn’t perform differently from those not in the program Health workers were disrespectful and asked for proof of menstruation for females when they asked about pregnancy prevention There was lack of privacy and a lack of adequate information provided to both genders More male MCs felt they received good quality care compared with female MCs
**Tanzania**; Mchome, Z. et al., 2015 [29]	Assess youth friendliness of reproductive health services in 2 regions in Tanzania (Mwanza and Iringa) Evaluated 8 workers from health centers and 25 workers from dispensaries	6 MCs had digital recorders in their pockets and underwent debrief interviews organized by a checklist and conducted by research staff and digitally recorded Data analysis: transcribed text analyzed for theme by a qualitative research team	6 male and female MCs that looked 18–19 years old assessed provision of care based on three scenarios: a condom and information request, information on STI’s after having unprotected sex a few days earlier, and a 16 year old girl asking about FP because she is feeling pressured to have sex with her boyfriend of six months Training consisted of becoming comfortable implementing the scenarios	Key findings included hostile or unwelcoming reception and dismissive staff saying SRH should only be for adults Reported lack of privacy and confidentiality MCs expressed how facilities lacked reliable opening times and had unexpected fees MC’s were happy to perform tasks, and be involved in future studies The MC method proved to be successful in evaluating the services
**Nigeria**; Ekong, I., 2016. [30]	Assess the adequacy of healthcare services for adolescents in southern Nigeria Assessed providers at health centers, adolescent service delivery centers, and hospitals in Akwa Ibom State	Qualitative and quantitative methods- surveys administered to MCs and questionnaires to providers in health center/hospital/adolescent health service delivery centers MCs interviewed directly after their visit by the researcher, audio recorded and analyzed manually	Female MCs evaluated reception, services to adolescents in health centers/YF centers MCs came with a written guidance note to ask for information about menstrual and other related problems MCs trained for an hour to prepare them for their role in the visit and to minimize potential disappointment from their experience	MCs reported relatively good communication with workers at all centers Some negative experiences were characterized by a lack of privacy and confidentiality during the consult and improper attention provided by the worker such as getting yelled at Office atmosphere and delays also contributed to poor experiences
**Ghana**; Pathfinder International, 2005 [31]	To assess factors hindering young people from accessing SRH care and to improve the quality of care Assessed 14 clinic heath workers, 10 Christian Health Association Ghana (CHAG) facility workers, 4 Planned Parenthood Association of Ghana (PPAG) facilities, and 10 other CHAG facility workers in Ghana	MCs and facility assessments were part of the data collection methods MCs filled out reports following their visits on their observations and evaluations on the youth friendliness of the clinics and client satisfaction Data analysis: reports compared and analyzed along with trends and client satisfaction data	MCs aged 15–24 observed their experiences regarding location of the health service, facility environment, staff preparedness, services provided, educational activities, peer education and youth involvement, administrative processes, and the cost of the service Training through lectures and role-playing involved covering the concepts of MC methodology, youth friendliness, adolescent reproductive health rights, essential package of YFS, and how to compile MC reports.	MCs found the clinics and facilities to be clean, easy to find, and with an adequate amount of information on the walls MCs noted challenges with health workers including negative treatment by providers and a lack of appropriate time and educational information given by the health provider MC method allows for the identification of both strengths and weaknesses of the service delivery
**Uganda**; Ndyanabangi, B., & Kipp, W., 2001 [32]	To generate data to enable development of RH programs in Kabarole to better meet the needs of adolescents Assessed staff in reception areas and service providers in the government hospital family planning clinic, health workers at a health center in a smaller township, and workers in commercial condom outlets	Used MCs in addition to key informant interviews with opinion leaders of the community including chiefs, ministers, and teachers MCs instructed to return for an interview after their session Data analysis: thematic analysis	2 male MCs went to health centers and shops to try and buy condoms 2 female MCs went to family planning clinics in government health facilities to buy OC Training consisted of preparation for their roles and minimizing potential harm during their experiences	MCs generally had good experiences One female received hostility while no males reported her that experience In the health center, the females received condoms without any information or counselling on how to use them In one health center, a female asked for OC but the nurse at the FP clinic warned the pill would ruin her reproductive organs and refused to give it to her All clients were asked their age by the shopkeeper when buying condoms The MC method provides unique information on assessing provider behaviours
**Uganda**; U. Arshad and B. Busingye, 2014 [43]	To assess whether 10 US funded HIV programs in Kampala, Uganda such as PEPFAR are upholding LGBTQ rights or creating barriers to HIV services for YMSM and LGBTQ youth	MC telephone calls and site visits were one component of the study MCs gave an overall rating out of 100% to the surveyed organizations	MCs visited sites at 10 organizations and made phone calls No other information provided	5/10 sites had mechanisms in place to reach YMSM and LGBTQ youth services but lacked some information on how best to do so Some instances of inappropriate questions and judgmental behaviour The waiting times were long without a designated youth waiting area and no tailored YMSM or LGBTQ youth information or education material
**Uganda**; Nalwadda G et al., 2011 [36]	To assess the quality of contraceptive services in different types of health facilities for young people aged 15–24 in two rural districts in Uganda, Mityana and Mubende, in order to identify areas of improvement Assessed 128 health care providers at public, private not-for-profit, and private for profit health facilities.	Guided by a theoretical framework to assess: technical competences of providers, information given to users, choice of contraceptive methods provided, interpersonal relations, continuity mechanisms, and appropriate constellation of services or appropriateness and acceptability MCs were interviewed with a structured questionnaire to address all quality of care variables Data analysis: descriptive statistics and factor analysis were performed using STATA V. 11	Two male and five female MCs, aged 15–24, were recruited from the two districts through youth leaders MCs were either graduate midwives or had advanced secondary education MCs had good communication skills and were fluent in both English and the local language MCs trained for three days using six case scenarios and role-plays that represented the main contraceptive methods Each female MC was given one scenario while each male MC was given two scenarios MCs systematically assigned to facilities and visited the health care providers to request contraceptive services	The quality indicators point toward limited compliance by providers with quality of care norms in contraceptive services Overall, the quality of care was low but also differed by facility type. Slightly higher total quality indicators were noted among public facilities compared with private-sector
**Benin**; Ambegaokar, MA., 2003 [17]	To assess the effectiveness of contraceptive technology training of private sector pharmacy staff in regards to their performance compared to non-trained pharmacy workers Assessed 72 private sector pharmacists in 9 localities of Benin-Cotonou, Porto-Novo, Abomey, Bohicon, Come, Igolo, Lokossa, Ouidah, and Sakete	MCs were one part of the method MCs assessed the performance of the pharmacists when they were providing information to a new contraception user and prescribing the pill Directly after the visit, the MCs completed an observation sheet on the things the provider did and said 3 MCs visited each pharmacy Data analysis: an evaluation team coded the observations made by the MCs	12 female MCs assessed providers’ performance when presenting a scenario of a young women who wanted to prevent pregnancy, but has never used any contraception and doesn’t know a lot about it All MCs were trained for the same scenario	Trained pharmacists performed better than untrained ones Majority of pharmacists (trained and untrained) received MCs courteously and privately MCs were given enough information on the pill, but were not provided with sufficient information on other contraception options and were not asked which method they would prefer Eligibility criteria for the pill were also not discussed Trained pharmacists did discuss how to use the pill and what to do if issues arise, and prescribed a low dose pill compared with untrained ones Reported that using MCs has fewer biases than other methods of quality assessment
**Nicaragua**; Henderson, B. USAID, 2003. [20]	To evaluate the performance of pharmacy workers in providing contraceptive counselling and services to adolescents Assessed pharmacy workers in 3 of the 5 RxGen target areas in Nicaragua	After pharmacy visits, the MCs were debriefed with questionnaires No information on data analysis was provided.	Both male and female MCs assessed pharmacy workers on whether a variety of choices of reversible contraceptives were presented, whether information given was accurate and available, and whether the environment was ‘youth-friendly’ with good communication, friendliness, and trust Training involved rehearsals of possible patient-client scenarios and instruction on observation and memory techniques	The majority of MCs reported good interactions with pharmacy workers; however some clients noted providers’ dismissive attitudes towards them They also noted a lack in written educational material provided MCs proved to be an effective method in evaluation despite extra effort required in training
**Nicaragua**; Meuwissen, L. E. et al., 2006. [34]	Assess whether a free adolescent voucher program in Managua- provided free ASRH care in public, private, and NGO facilities and trained providers on best practice protocols and guidelines- changed in regard to technical quality, communication and treatment provision towards adolescents Evaluated use of MCs as a method of assessment Assessed health workers at 19 different clinics – 10 NGOs, 4 public and 5 private health facilities	Clinics were evaluated pre- during- and post intervention MCs both with and without vouchers visited clinics MCs interviewed after visits by a doctor on the research team with a standardized questionnaire focusing on 21 criteria including the method of contraception provided, explanation of advantages/disadvantages of the method, and the organization of the visits A clinic score was calculated based off the questionnaire completed by the MCs Data analysis: Epi-info and Stata	17 female MCs aged 16–22 Presented a story where they were in a relationship in which they were using withdrawal or periodic abstinence, but wanted to avoid pregnancy and wanted information on HIV Training consisted of explaining the purpose of the study, reviewing their roles, and educating the MCs on how to not be physically examined	The majority of MCs received information on all contraception options Reliability of information varied- some doctors advised against condoms because they were unsafe and could lead to allergic reactions Most patients reported they felt the providers were in a hurry during their consult Patients with a voucher were more likely to make a joint decision on contraceptive method with their doctor (p = .02) compared with those without a voucher having the doctor make a decision on their behalf Female doctors had higher scores than male doctors Differences found using the MCs are considered indicative of real differences in provider performance
**Mexico**; Wolfe, K., 2005 [16]	To assess how well a program designed to train pharmacists and provide support and materials for YFS improved quality and YF of services compared to control sites at pharmacies in Guanajuato, Mexico 100 visits were made to pharmacy workers and staff in pharmacies	MCs were part of the methodology which included pre/post training examinations of pharmacy providers, surveys on the health needs of adolescents, and a school-based survey on reproductive health, and focus groups of youth, pharmacy staff, teachers, and parents. MCs visited control and intervention pharmacies After their visit, MCs filled out a survey on their experience and interaction with the pharmacy staff No information on data analysis was provided	Male and female MCs aged 15–19 Evaluated provider behaviour and the information provided to youth clients while using one of four scripted scenarios (not detailed in the report) inquiring about contraception and other reproductive health inquiries MCs observed the information provided, the workers’ behaviour, and usefulness of counsel provided MCs were trained and prepared to do a pharmacy visit and complete the survey for evaluation thereafter	Trained pharmacists provided better SRH/FP information than control pharmacists Trained pharmacists also provided a more comfortable and confidential environment, showed more interest in clients' health, and were more likely to provide condoms and information on their use to MCs
**Mexico**; Clyde, J. et al., 2013 [33]	To assess whether regulations and clinical attitudes and practices hinder girls’ (12–17) access to pregnancy termination and abortion services in Mexico City Assessed health workers at 11 public hospitals and 3 NGO facilities	MCs were part of a methodology that also included focus groups and interviews with adolescents who had unwanted pregnancies, surveys for abortion clinic directors/NGO staff, and interviews with adolescents after seeking abortion services MCs were debriefed by research staff from an interview guide by IPPF/WHR and an Investigacion en Salud y Demografia (INSAD) team focusing on organization of the visit, staff treatment and reactions, and access to information/services Data analysis: excel matrix and SPSS	Four female MCs trained to assess barriers to accessing quality and comprehensive abortion services An adult accompanied some MCs while others were alone One MC was pregnant MCs observed staff and assessed support for adolescent decision-making Trained to play the role of women seeking abortion information and counselling	MCs received mixed information on whether adolescents need to be accompanied or not by an adult Several providers supported autonomous adolescent decision-making For accompanied MCs, health workers did not ask the majority of clients if they wanted to be talked with alone, leading them to make different decisions then they would have alone MCs tended to receive more information when accompanied by an adult
**Bolivia**; Belmonte, L.R. et al., 2000 [38]	To evaluate the physical, psychosocial, structural, and economic barriers for adolescents in the use of RH services in 3 Bolivian cities (2 health facilities per city in La Paz, El Alto, and Santa Cruz)	MC’s were part of a set of qualitative and quantitative methods including participatory learning- a ‘social mapping’ exercise, surveys given of adolescents, focus groups of adolescents in and out of school, and interviews with health service providers and directors of health facilities, and MCs MCs were debriefed after their visits No information on data analysis was provided.	MCs assessed service provision and barriers they perceived when they went to the health facilities No information on training provided.	Providers expressed more disapproval providing contraception to girls compared with boys Providers many times gave the wrong information- like girls rarely get pregnant the first time they have sex There were two instances of inappropriate behaviour where doctors tried to seduce female MCs Females also felt they were being ‘preached to’ Physical barriers were not an issue due to location of facilities in big cities
**Egypt**; D. Oraby et al., 2008. [40]	To assess the YFCs of teaching hospitals and how effectively they deliver comprehensive RH care in Egypt Assessed health providers in eight UNFPA supported YFCs in teaching hospitals – El Galaa, Shoubra, El Mataryea, El Sayeda Zeinab, Banha, Shebin El Kom, Damanhur, and Sohag	MC’s were part of a methodology that also included interviews with service providers on their perceptions of clinic operations, client exit interviews, and reviewing project documents such as service statistics and program records MCs evaluated location of clinics, patient flow and organization of clinic, staff and health provider behaviour and interaction, and satisfaction with the service After their visit, the MCs reported their findings to the study team No information on data analysis was provided.	MCs assessed the services based on location, the clinic environment, their interaction with service providers, and their satisfaction with the service MCs were to go to the reception of the outpatient center of the teaching hospitals and ask to be guided or brought to the YFC Once there, the MCs were to discuss a fictional RH complaint while observing the physician interaction If offered laboratory services they were to accept it as well MCs were given information on the location of the services, the services provided by the YFCs, and informed to seek advice on a complaint relevant to an RH service	A doctor was readily available for MCs at only one of the facilities; the majority of clients were unable to see a doctor and were told to return the next day, but were treated respectfully Finding the YFC was difficult even for some MCs within the hospital having asked at reception
**India**; Santhya, K. G. et al., 2014. [37]	To examine access and components of ASRH care from the perspective of adolescents and health care providers in three states in India Assessed health providers at 12 Adolescent Friendly Health Centres (AFHC’s) in rural areas in 3 states- Jharkhand, Maharashtra and Rajasthan	Mixed methodology MCs, surveys of youth in community settings, exit-interviews of young clients accessing services, and in-depth interviews with ASHAs, ANMS, MOs, and counselors The study supervisor debriefed MCs after their visits using a questionnaire focusing on the registration process, wait time, provider interaction, information provided during the consult, provider behaviour, and their comfort level during their visit The MC and paired observer (also present during the visit) were debriefed separately using a questionnaire Data analysis: survey data was analyzed using SPSS/STATA and the in-depth interviews were recorded, transcribed, and thematically analyzed	24 male and female MCs aged 18–24 were to observe and evaluate everything the health care providers did and said during their visits They were to execute one of four scenarios: a 17 year old female seeking information on contraception because she feels pressured to have pre-marital sex; a 20 year old female seeking protection because her husband has other sexual partners; a 19 year old male seeking condoms; a 22 year old male wanting an HIV test after having sex with a sex worker MCs visited the health centers in pairs Trained by study coordinators on the scenarios via role-play activities and learned how to evaluate the\services	All MCs received some information on contraception but explanation on use and different options varied Not all were given contraception and those who didn’t receive it weren’t given a proper explanation MCs noted overall positive experiences but mentioned issues with privacy, comfort, and some providers made judgmental comments including saying they would only provide condoms if the female client’s husband was present and that it was wrong for young people to have sex before marriage All of the MC scenarios resulted in providing information on contraceptive methods offered at the health centers
**India**; A. Barua and V. Chandra-Mouli, 2016 [41]	Evaluation of the Quality of AFHS in the TARUNYA – ARSH project; Jharkhand	MCs were trained by WHO consultants in appraisal of the facilities, and attitudes and behaviours of providers to adolescents presenting with SRH conditions	4 MCs, 2 adolescent girls and 2 adolescent boys were trained to present SRH scenarios to health care providers in government secondary health facilities in Jharkhand	In most facilities, infrastructure and ambience was adolescent friendly, but educational materials were not adequate in some Service providers greeted the MCs, maintained confidentiality, listened with attention, were considerate to their needs, gave addition education However referral was not adequate (probably because this was a secondary facility with in house departments) For cases where services were refused it was seen that MCs came in for SRH services while regular adolescent clients came more often with general medical problems
**Bangladesh**; Sarma, H. and Oliveras, E., 2011 [45]	To evaluate the effect of a program that trained private pharmaceutical company medical representatives to distribute STI guidelines and counselling services to 6 different non-formal providers in intervention areas compared to areas without the intervention in Bangladesh.	MCs were debriefed on their visit via a checklist that focused on provider behaviour and interaction with the patient, treatment advice provided, and information on condom use and partner management Data analysis: SPSS software	24 male MCs aged 15–24 evaluated provider behaviour toward STI clients, advice given on treatment, condom use, and partner notification Training was one day long covering how to act like a MC, how to avoid physical examination, and information on how to evaluate the service after their visit.	Majority of MCs experienced friendly service Poor experiences consisted of providers not giving adequate advice and information on condom use and partner management
**Tonga**; L. Havea, 2007 [39]	To assess the way in which provider behaviour and clinic characteristics impact young peoples’ utilization of RH services and determine the level of utilization of ASRH services in Togatapu Assessed RH service providers in governmental health clinics in the Kolomotu’a, Vaini, and Kolonga Districts, and in the NGO TFHA Clinic of Topodatapu, Tonga	Mixed methodology was used including interviews of service providers on youth friendliness of services, MCs, focus groups with young girls and boys and users of the TFHA services, and data reviews on inventory and services provided by facilities Five MCs visited each site MCs filled out questionnaires and had exit interviews on their impressions outside the clinic after their visit in addition to providing a verbal summary Data analysis: Epi-info and thematic analysis	7 female and 8 male MCs who were all younger than 25 Assessed clinic reception, provider communication, advice given, and their ability to discuss clients concerns while carrying out one of four scenarios: seeking contraceptive services, seeking STI treatment, being concerned about a possible pregnancy and requesting a pregnancy test, and seeking counselling on the EC pill MCs were trained to visit the clinics using the four scenarios	Majority of MCs reported being comfortable and having sufficient privacy MCs reported that providers gave their opinion on what they should do Despite providing information, some providers didn’t check with MCs to see if they understood the information Some providers made the MCs feel uncomfortable, acting as if they were not taking the MC seriously or laughing at them MCs were shown to be a good tool in giving feedback on provider behaviour during the provision of care to young clients If they are well prepared it is quick and provides good feedback, which is important for improving services The lack of training of some service providers was confirmed through the analysis of the MC’s findings
**China**; Institute for Planned Parenthood Research on behalf of the China Youth Reproductive Health Project, 2005 [35]	To assess service delivery and utilization of the Youth Friendly Service Centers in Shanghai Assessed RH workers and staff at 1 city level health center, 1 population and family health website, 2 adolescent activity centers, 3 middle schools, and 2 community family planning service stations	Mixed methods: MCs, interviews with 11 health managers and 12 health providers, facility inventories, observation of communication between providers and adolescents, and reviewing data and records on the use of services, quality of care, and the adolescents’ current needs for service MCs filled out an observation form the day of their visit, which provided data for the evaluation framework based on 17 indicators including management, staff, facilities, and atmosphere of the facilities An overall score was calculated to illustrate youth friendliness of the center	3 female and 3 male MCs Observed convenience of location, waiting time, hours of facilities, the privacy, competencies and attitudes of providers, peer education from staff, policy support, and gender equality in service Via in person, online, and hotline counselling methods. 1 of 4 scenarios was employed: having an unwanted pregnancy, wanting information on contraception, seeking information on STIs, and seeking counselling on pre-marital intercourse Training consisted of becoming comfortable with the scenarios.	Some centers were reported to provide consults with privacy There seemed to be an insufficient number of fulltime, skilled service providers, limiting availability and access There were also inconsistent opening times
**South Africa**; Mathews et al, 2008 [42]	To evaluate whether HIV testing services in clinics involved in an adolescent-friendly initiative were of higher quality than clinics not involved is the initiative	Used MCs in 12 clinics involved in the National Adolescent Friendly Clinic Initiative and 2 clinics that were not in the initiative MCs included adolescents who knew their HIV status and were recruited as MCs to seek HIV tests from the clinics	Two adolescent MCs were HIV positive and all were “black” Xhosa speakers Adolescents were between 16 and 19 years old 4 male adolescents and 6 female adolescents Adolescents received 10 hours of training and were screened by a psychiatric nurse to determine if they would be negatively affected by participation in the study Interviewed by a researcher with a questionnaire within 2 hours of the HIV test at the clinic Adolescents were reimbursed for each clinic visit that resulted in an HIV test	Improved accessibility to HIV testing No impact on adolescent’s experience of negative attitudes from health workers and confidentiality breaches Adolescents less likely to be dismissed without a test at NAFC clinics but confidentiality breaches were prevalent in both categories of clinics due to similar clinic setups- lack of privacy and batching patients for blood sampling. Value of MC use- real clients would not want to criticize service providers or be a part of research after a positive HIV diagnosis
**Thailand**; Ratanajamit et al., 2008 [25]	To evaluate the effectiveness of an intervention to improve knowledge and delivery of EC among drugstore personnel	Randomly selected 60 drugstores in hat Yai; half assigned to participate in an educational program MCs went into the stores before the intervention and at 1 and 3 months after the program	3 male and 3 female MCs age 20 to 22 randomly assigned to different phases of data collection MCs did not know the intervention status of the assigned drugstores Scenario: college student had unprotected mid-cycle intercourse and wanted to prevent pregnancy	Intervention group improved significantly in dispensing and advising about EC after training Knowledge that EC should be initiated within 72 hours improved with the intervention group History-taking practice was not improved by the training Stated EC to be a sensitive issue and therefore is dispensed quickly without asking questions about sexual history

Within these settings the evaluations/studies studied health professionals (doctors, nurses etc.), reception workers, and pharmacists.

### What were the principal findings from methodology reported in the evaluations/studies?

While the principal findings of the reviewed evaluations/studies varied, key themes emerged.

#### Friendliness of health care facility

The majority of MCs in the evaluations/studies reported overall good experiences with friendly staff. One particular study scored the friendliness of the services in the various facilities in their evaluation as 73% [27]. Some other good practices were reported by the MCs, such as the willingness of the facilities to provide services for young people, the provision of services for LGBTQ youth [26,43] and the provision of information (although not always adequate).

Importantly, MCs also reported inappropriate behaviour or inadequate service provision that lead to poor experiences. From the 30 evaluations/studies, eight found MCs to have had issues with the lack of privacy and/or confidentiality provided by the health care providers during their visit [18,27–30,34,37,42,44]. For example, one MC expressed that,

“It wasn’t great, it wasn’t private, and you had a discussion in the open. There were other people there and it could have been really awkward… Overall it wasn’t a good experience” (Mystery Client, Nottinghamshire, England) [28].

MCs also observed structural barriers including location, opening times, and staff shortages of health facilities in eight of the reviewed evaluations/studies (26.6%) [18,19,29,31,35,37,40,43]. Staff shortages and limitations in opening times were reported to impact accessibility to services in the reviewed assessments. In the following case, a 19-year-old MC acting as an unmarried young man in a relationship with a girl and seeking condoms reported the following;

“The doctor advised me to use condoms but he told me that he wouldn’t be able to give them to me that day because the store was locked and the designated staff member was on leave.” (AFHC C, Jharkhand) [37].

In four studies, MCs described sessions with health care providers to have been rushed, as the providers did not spend an adequate amount of time with them [24,30,34,37]. They reported health care providers to be hasty or preoccupied during their visits, which impacted the focus on the MC’s needs, provide enough information, or fully and adequately answer their questions or concerns. A study in Thailand indicated that because EC is a sensitive subject in the country, it is often quickly dispensed and pharmacists do not spend time asking the necessary questions related to sexual history (49). In addition, an evaluation in Nicaragua reported that;

“More than 10% of the SPs (simulated patients) in each round felt the doctor was in a hurry to end the consult. Some felt pressured to the extent that they did not ask their question on STIs” (Nicaragua) [34].

#### Provider behaviour

In 14 of the reviewed evaluations/studies (46.6%), MCs experienced inappropriate or disrespectful behaviour towards them during their consultation [18,20,26–31,37–39,43,44]. Inappropriate behaviour ranged from seductive behaviour to being laughed at and not taken seriously. In most cases, the disrespectful behaviour consisted of judgmental comments made by health care providers on the inappropriateness of young people to seek SRH services. In the following case, a MC experienced judgemental comments regarding her lack of marriage status.

“… When I started to explain my need of FP methods s/he asked me twice, Family planning? So, are you married?” (Mystery Client, urban-intervention ward, Mwanza) [18]

Some of the reviewed evaluations/studies described differences in provider behaviour and attitudes based on gender of MCs. Some reported that male MCs were more likely to report services as satisfactory compared to female MCs [32,38,44]. The female MCs in these evaluations/studies tended to report attitudes and behaviours that were inappropriate and affected their experience at the facilities they visited. For example;

“…in two cases, physicians tried to seduce the (female) mystery clients. Other inappropriate behaviour included aggressive tactics and inappropriate intimacy used by the providers” (Bolivia) [38].

One study reported findings that differed from this pattern, stating that young male clients face more difficulties when seeking contraceptive services in rural Uganda [36]. Ultimately, although most of the evaluations/studies did not clearly state differences between the male and female MCs’ satisfaction with services, it appears that female MCs reported more hostile attitudes from the service providers than males.

Additionally, two evaluations/studies mentioned lack of decision autonomy to be problematic during MC sessions [33,34]. In these cases, the health care provider made decisions for patients without consulting their opinion:

‘She said I should take the injection and that we shouldn’t go into the other methods. When I asked why she recommends the injection she asked how old I am and said that they don’t recommend pills for young people because they are careless…’ (Simulated Client 5 (female), Clinic 3 – YFS, Benin) [17].

In another study, when the MCs were accompanied, decision making autonomy was restricted because health care providers did not offer the adolescent an opportunity to talk without the accompanying person [33]. The authors of this study link this to the fact that a portion of the adolescents ‘ended up with a decision about abortion that they did not initially prefer’ [33].

#### Information availability

Availability of information and quality of available information was a concern in many of the evaluations/studies. This included information from verbal communication during sessions with health care providers and/or information in printed format, such as posters or pamphlets. Fourteen of the reviewed evaluations/studies (46.6%) indicated that MCs found the information provided by the health care providers to be insufficient [2,5,6,13,17,20,23,26–31,33,36,37] The following quotes, from studies in Uganda, Mexico, and Nicaragua, indicate examples of lack of quality information provided;

“…none of the organizations visited had tailor-made targeted information, education and communication (IEC) materials for YMSM and LGBTQ youth” (Uganda) [36].

“…there were cases in which professionals either gave incorrect information (stated that the procedure had to be surgical because the girl was an adolescent or said that the reason an accompanying adult was required was because the procedure was very risky) or made specific judgmental statements that appeared related to the adolescent’s age” (Mexico City) [33].

“Pharmacies tended not to display written or visual information, and few had fliers or other written materials to take away” (Nicaragua) [20].

### What were the overall perceptions of the use of MCs in assessing health care provider and facility performance?

Generally, the MC methodology was perceived to be a useful method in evaluating service delivery and provider behaviour. Twelve of the 30 evaluations/studies explicitly stated perceptions or observations on the use of adolescent MCs in evaluating health provider behaviour and service provision [17,20,28,29,31–34,37,39,41,42] Of these, three regarded MCs as being a good method for providing insightful and unique feedback [28,32,39] and six indicated that the use of MCs was successful in assessing services and providers [20,29,31,34,37,42].

Additionally, one study discovered that MCs accompanied by adults received more information than those that were unaccompanied [33]. Similarly, in one study adult physicians received correct information and more attention from pharmacists when calling in regard to adolescents’ access to EC than the adolescents themselves [24]. Also, in instances when services were refused, MCs had reported at the facilities with SRH service needs, whereas the actual adolescent clients presented with general medical issues [41].

The reviewed evaluations/studies pointed to the need to invest effort in training and preparing the MC. This was seen as crucial to the success of the evaluation or research study [20,39] Other evaluations/studies found the MC method to be a quick way to receive feedback, critical for improving services, and that the conclusions from these evaluations/studies could be extrapolated to the general population because the MCs’ experiences represented actual treatment of adolescents within the healthcare delivery system.

## Discussion

Overall, the reviewed evaluations/studies indicate that the MC methodology is widely used and valuable in the examination of health facilities and health care providers. First, we found that the reviewed evaluations/studies used the MC methodology, either alone or in combination with other methods, to obtain a youth observatory perspective of how the health facility or health care provider responded to providing SRH needs to adolescents. Secondly, in some evaluations/studies, the roles of the adolescent MCs were well defined, while others only provided limited detail. Also, some evaluations/studies described greater preparations such as training, carefully detailing scenarios, and role playing. Thirdly, the methodology was used to examine adolescent health service provision in public and private hospitals, NGO supported facilities, formal and informal settings, and pharmacies. The cadre of health care providers evaluated or studied with MCs varied and included health workers (doctors, nurses, etc.), pharmacists, and reception workers. Finally, MCs requested a variety of information on testing and treatment of HIV/STIs, menstruation issues, abortion services/options, unwanted pregnancies, contraceptive services, and other SRH issues. To conclude, this review confirms that while adolescents in high-, middle-, and low-income countries tend to have positive experiences in SRH services, they sometimes experience judgmental and disrespectful behaviour from health care providers or inaccurate/insufficient information from them and find that ASRH services may not be organized to their needs and preferences.

Despite concerns that the methodology limits the type of information collected, considering that MCs do not undergo any form of medical examination or procedure, researchers are still able to draw valuable information from MCs’ experiences regarding facility performance and provider behaviour. By using mixed methodology and properly trained MC participants, it is possible to increase reliability and address some of the pitfalls of the methodology and provide additional insight into areas of interest [1,35,39,46]. Most of the evaluations/studies reported debriefing the MCs immediately after their visits to avoid recall bias. Each report indicated different methods to debrief MCs, but all debriefs were carried out immediately after the visits. Although previous studies have regarded MCs as more effective in monitoring, compared to evaluation [1,39], the reports included in this review prove otherwise with insights into how the method can be used and adapted to suit the purpose of the evaluation. Although we cannot say that this methodology is better than other qualitative methods, as there was no comparison of methods, we can draw from the experiences of the researchers and evaluators who found it effective in providing a glimpse of information on health facilities and health care provider behaviour. Two key advantages of the MC methodology include its potential for quick feedback, and its association with reduced incidence of bias, such as observer bias and bias from Hawthorne’s effect.

The evaluations/studies in this review describe similar findings to other evaluations/studies that have reported on health care provider behaviour and SRH facilities and services with different methods. Gonsalves and Hindin’s review confirms that both pharmacists and young people are concerned about access to SRH services and recognise that regulations can increase barriers to this access [4]. Our review also aligns with a recent review addressing the barriers that adolescents face in accessing STI services; behaviour was found to be satisfactory for the most part, but included instances of inappropriate and disrespectful conduct towards adolescents [47]. Newton-Levinson et al.’s review also suggests that there might be differences in the attitudes of health care providers depending on gender of the patient/client and that female adolescents may perceive female health care providers as being friendlier than male health care providers [47]. Similarly, an article addressing adolescent SRH needs from an evaluation in Uganda reveals that SRH services sometimes do not provide a safe environment for adolescents to discuss sensitive issues and that this affects the patients’ ability to feel comfortable to fully disclose concerns and issues [48]. Our review of the MC methodology with adolescents provides more depth on SRH services from their unique perspective. The review indicates that in addition to the lack of assured privacy and confidentiality, the timing constraints of services, recurrent staff shortages, and unexpected costs of services or commodities are key barriers to adolescents’ access to SRH services. Further, the MC methodology is as good a method for M&E because it identifies both strengths and weaknesses of health service delivery and provides the unique perspective of the MCs, the people for whom the service is designed.

The results and experiences of the MC methodology with adults and in the business sector [5–8] are similar to the results of our review regarding adolescents in the health sector, and thus add support to the value of this method. For example, in an evaluation study by Friedman et al., MCs were trained to assess provider practices and evaluate the use of mobile phone messages to pharmacists to improve the treatment of childhood diarrhoea in Ghana [49]. The study found disparities between actual practices of the providers and their self-reported practices. Outside the field of health, the MC method has evaluated the quality and quantity of information provided by the staff of financial institutions in Ghana, Mexico, and Peru [8]. The study found that clients were not offered enough information to compare credit and savings products [8], similar to the result in which we found that adolescents were not offered enough information to compare contraception methods [17]. The efficacy of the MC method in ASRH service provision is supported by the success of these studies and the similarities in results and experiences.

The MCs in our reviewed evaluations/studies reported positive experiences, similar to the perceptions of adolescents involved in other participatory research methods. For example, in Nigeria, adolescents used the photovoice technique, which involves taking photos of issues within their communities to then critically discuss, and reported that they enjoyed the experience and would be willing to participate in future research evaluations/studies [50]. The MC methodology provides an opportunity for the clients to be engaged in their own care and services. In some cases, MCs also receive training in other areas such as evaluation techniques and receive education on SRH issues. Furthermore, we can draw from the reports and comments of the MCs and suggest that they would share their knowledge and experiences with their peers. The positive response to the MC methodology, from adolescents themselves, contributes to the merit of the methodology.

Despite the expansive search undertaken in the review, some evaluations/studies may have been missed. Our findings are limited by our criteria, that excluded reports not published in English or Spanish and our inability to find similar reviews in the Spanish language which impacted the geographical distribution of evaluations/studies. No quality assessment was undertaken, which limits our ability to assess and integrate those quality evaluations into our findings. Some of the evaluations/studies used very few MCs or examined a small array of healthcare providers and services, which could impact their findings. Furthermore, several evaluations/studies were unclear or did not include information on several components of their study, making it difficult to accurately synthesise findings. The evaluations/studies that used multiple evaluation techniques in evaluating provider behaviour and service provision did not directly compare MCs outcomes to those of other forms, which limits our ability to understand the extent to which the MC assessment was effective, compared to other methods. The review did, however, cover a breadth of research designs in a variety of settings, allowing for a comprehensive analysis.

## Conclusion

The use of MC methodology with adolescents provides an opportunity for researchers and evaluators to understand how health care providers and health facilities perform when they do not know they are under review. It is a useful M&E method that allows for quick feedback to identify how to improve the quality of ASRH services. The use of adolescent MCs, in particular, aligns with the call for the participation of young people in programme and policy development [6] and the need to champion adolescents’ SRH rights. The findings of this review could help inform the decisions that policy makers and programme developers make by improving their understanding of the SRH needs of adolescents. The successful and effective participation of adolescents and young people as MCs demonstrates their ability to actively participate in information gathering and research analysis for work that will ultimately benefit them. When given the chance, young people can be more than just subjects in research or evaluations, and instead become meaningful participants.

## Paper context

Mystery client (MC) methodology is a form of participatory research for evaluating the performance of health care providers or facilities from the perspective of service users. Until this review, there were no systematic reviews on the use of MCs in adolescent sexual and reproductive health (ASRH) research and monitoring and evaluation of programmes. This review highlights the usefulness of MC methodology to gain insight, from an adolescent perspective, on the quality of ASRH services.
